# Notes on Medical Schools and Colleges

**Published:** 1906-09-01

**Authors:** 


### NOTES ON MEDICAL SCHOOLS AND COLLEGES.

### LONDON.

### St. Bartholomew's Hospital Medical School.

St. Bartholomew's is the oldest and second largest hospital in London, and is, perhaps, the leading medical school. The site has been recently enlarged by the purchase of $1\frac{1}{2}$ acre of adjoining ground, and the first block of new buildings is in course of erection at a cost of £120,000. It will contain new casualty and out-patient departments, eight special departments, new quarters for the resident staff, a dining-hall and common room for students, etc. Two additional operation theatres have been built, making seven in all. The erection of this block will not interfere with the work either of the hospital or the school.

### The Charing Cross Hospital Medical School (The University of London).

The winter session will be opened on Monday, October 1, by the delivery of the Sixth Biennial Huxley Lecture on "Recent Advances in Science and their Bearing on Medicine and Surgery," in the Anatomical Theatre of the Medical School, at 4 p.m., by Professor Ivan Petrovitsch Pavloff, of St. Petersburg.

Situated in the very centre of London, this hospital has perhaps an advantage over all other similar institutions in the metropolis as regards accessibility.

Extensive additions have recently been made, including new surgical wards, out-patient department, operating theatres, and isolation wards.

The medical school occupies a separate building in Chandos Street, immediately opposite to the hospital.

(1) Composition fee for medical students, payable in one sum on joining, 115 guineas. (2) Sessional payment system: Entrance fee, 10 guineas. In addition a sum of 15 guineas must be paid at the beginning of every winter session, and one of 10 guineas at the beginning of every summer session so long as the student remains in the school.

The fees for dental students for the two years' curriculum required by dental students may be paid: (*a*) in one sum of 55 guineas on entry; (*b*) in two instalments—one of 31 guineas on entry, and the second of 30 guineas at the end of the first twelve months.

Full information respecting the medical school, and the course of study recommended, may be obtained on application to the Dean, Dr. Christopher Addison, at the Medical School, Chandos Street.

### St. George's Hospital Medical School.

The hospital contains 350 beds, of which 205 are devoted to surgical and 146 to medical cases.

There are several entrance and other scholarships.

The entire laboratories and teaching at the hospital and school are now devoted to the essentially medical subjects. Unequalled facilities are therefore available for clinical instruction and research.

There are numerous appointments open to the senior students, and amongst others may be specially mentioned the following paid appointments, which are open to students after they have held house office: Two medical registrarships, salary £200; surgical registrarship, £200; curatorship of the museum, £200; assistant curatorship, £100; resident obstetric assistantship, £50; two demonstratorships of anatomy, £50; senior anæsthetists, £50; two junior anæsthetists, £30 per annum.

### Guy's Hospital Medical School.

Guy's Hospital contains 602 beds, and a noteworthy feature is the reservation of certain wards containing 40 beds in all, for cases of special clinical interest. By paying an annual subscription of three guineas any student of the hospital obtains the privileges of membership of all the clubs, including the students' club.

The composition fees are 16 guineas for the first twelve months or less period, and subsequently 30 guineas a year till the student is qualified.

### King's College Hospital.

King's College Hospital, which contains 220 beds, is situated in Portugal Street, Lincoln's Inn, and is about five minutes' walk from King's College.

The new hospital, which is now being built in the neighbourhood of Denmark Hill, will be on a much larger scale than the present institution, and will possess every modern requirment for both patients and students. The teaching of the preliminary subjects, including chemistry, physics, biology, anatomy, and physiology, will continue to be given at King's College in the Strand, and students entering now may by the time they are sufficiently advanced to study in the wards be able to do so in the new hospital.

The composition fee is—for University course, 140 guineas; for conjoint course, 135 guineas. Full particulars and prospectuses can be obtained by applying to Professor Halliburton, Dean of the Medical Division of the Faculty of Science, or Dr. Whitfield, Dean of the Hospital.

### The London Hospital Medical College.

The London Hospital is the largest institution of its kind in England, and being situated in the East End, it ministers to a very large and poor district.

Seven entrance scholarships are offered annually, including the Price Scholarship in science, £120; the Epsom Scholarship, £126; and entrance scholarships in science, anatomy and physiology, and arts.

The composition fee is 120 guineas if paid in one sum, or 130 guineas if paid in three annual instalments. A reduction of 15 guineas is allowed in the case of sons of medical men.

Full information may be obtained from the Warden of the Medical School.

### St. Mary's Hospital Medical School.

St. Mary's Hospital at present contains 281 beds, which number will be raised to 341 on the opening of the Clarence Wing. This extension will also provide new operating theatres, a medical clinical theatre, an obstetric department for cases of difficult labour, and a complete electrical department. A new $x$-ray department has recently been opened in the out-patients' department.

The composition fee is £140 if paid in one sum on entering the Medical School, and £145 if paid in four instalments. On payment of the full composition fee, and provided that he obtains a registrable medical qualification within seven years from the date of registration, the student becomes a perpetual pupil, and is entitled to unlimited attendance on the practice of the hospital. The composition fee for students who have passed their examination in anatomy and physiology is 60 guineas if paid at entrance, 65 guineas if paid in two annual instalments.

### The Middlesex Hospital Medical School.

The hospital, which is in Mortimer Street, W., contains 340 beds, including special wards for children and the diseases of women. There is a cancer wing which has 40 beds, and special research laboratories, and the hospital therefore offers unequalled opportunities for the study of this disease, both clinically and pathologically.

A complete electrical and $x$-ray department has been provided in a temporary building which has been erected in the courtyard of the hospital.

### St. Thomas's Hospital Medical School.

St. Thomas's Hospital contains 602 beds, of which about 540 are in constant use.

The various students' clubs were amalgamated in 1888. The annual subscription for membership of the amalgamated clubs is £3 3s. per annum.

The composition fee covering the work of the first year is 16 guineas. Students who have passed the preliminary scientific or a corresponding examination pay an entrance fee of 20 guineas and an annual fee of 30 guineas, which is due in advance on the first day of the term in which the student enters, and on the corresponding day of each successive year until a registrable qualification has been obtained. A student who qualifies within 3 months of the date on which his last annual composition fee became due is allowed a rebate of 20 guineas, if within six months his rebate is 10 guineas.

Winter Session opens on Monday, October 1. This is the best date for a student to begin preliminary work.

Full particulars will be sent on application being made to G. J. Roberts, M.A., Medical Secretary.

**University College Hospital Medical School** comprises the departments of medicine and clinical medicine, surgery and clinical surgery, midwifery and gynæcology, pathology and morbid anatomy and clinical pathology, bacteriology, mental physiology, and mental diseases, dental surgery, practical pharmacy, and other departments for the study of special diseases, such as those of the eye, skin, ear and throat, and for instruction in the use of anæsthetics, and in electro-therapeutics and the application of the $x$-rays.

A student may enter the school as soon as he has passed the University of London Matriculation Examination or one of the other preliminary examinations that qualify a medical student for entering a medical school. In this case he will pursue his preliminary and intermediate studies at University College, and when those are completed will carry on his advanced medical studies at University College Hospital Medical School. The student who in addition to having passed a matriculation or other examination, has completed his preliminary and intermediate medical studies at University College or elsewhere, may enter the University College Hospital Medical School for his advanced medical studies only.

Scholarships and exhibitions to the value of about £450 are offered for competition every year.

Composition fees for the courses required by the University of London: For the Final M.B., B.S. course, 80 guineas, or in two instalments of 50 and 32 guineas; for the medical education required by the Examining Board in England and the Society of Apothecaries, for the course required for the third examination, 80 guineas, or in two instalments of 50 and 32 guineas.

### Westminster Hospital Medical School.

Westminster Hospital was the first institution of its kind in London which was founded by the voluntary contributions of the public. There is accommodation for upwards of 200 in-patients. There is a Students' Club Union, the subscription for membership of which is included in the general fees. The composition fee is 120 guineas for the course of the Conjoint Board, and 140 guineas for that of the University of London.

### London (Royal Free Hospital) School of Medicine for Women.

The Royal Free Hospital is in Gray's Inn Road, and the London School of Medicine for Women is in Hunter Street, Brunswick Square, W.C.

There are 165 beds in the hospital, all of which are available for clinical teaching.

There are numerous scholarships and prizes offered annually in connection with the school, among which may be mentioned the School Scholarship of £30 and the St. Dunstan's Medical Exhibition of £60 a year for three years, extendible to five years.

### PROVINCIAL MEDICAL SCHOOLS.

### The University of Birmingham (Faculty of Medicine).

Candidates are allowed to enter for the First Professional M.B., Ch.B. Examination (Chemistry and Physics) before commencing residence in the University, provided that they have already passed the Matriculation Examination or some preliminary examination accepted by the University in lieu of its matriculation, such candidates to be eligible for the final examination for M.B., Ch.B., after four years' residence in the University and by producing evidence that they have been registered medical students by the General Medical Council for a period of five years. In order to obtain the degrees of M.B. and Ch.B. five examinations have to be passed. The first is in chemistry, physics, and elementary biology; the second in anatomy and physiology; the third in pathology, bacteriology, and materia medica and pharmacy; the fourth in forensic medicine, toxicology, and public health; and the fifth, or final, in medicine, surgery, midwifery, gynæcology, therapeutics, mental diseases, and ophthalmology. The University also grants degrees in dental surgery, B.D.S. and M.D.S., and a diploma (L.D.S.), and a diploma (D.P.H.) and a degree of B.Sc. in public health. The General Hospital and the Queen's Hospital, containing between them upwards of 400 beds, are amalgamated for the purpose of clinical instruction, which is carried on under the direction of the Birmingham Clinical Board.

The composition fee for the whole course of lectures and instruction at the University is £85, besides which there is a composition fee for attendance on the medical and surgical practice and the clinical lectures at both hospitals of £42, making a total of £127. Dean: Professor Gilbert Baring.

### University of Cambridge.

In order to obtain the degree of Bachelor of Medicine a student must become a matriculated student of the University, reside in the University the required portion of each of nine terms, pass or obtain exemption from the previous examination, pursue medical study for five years, and pass three examinations.

Instruction in clinical medicine, surgery, and midwifery is given at Addenbroke's Hospital, so that it is possible to obtain a complete medical education at Cambridge; but the usual practice is for men, after spending three or four years at the University, and passing their examinations in anatomy and physiology, and in pharmacology and general pathology, to finish their clinical work in London or one of the large provincial towns.

### University College, Cardiff.

Since it was founded, in 1883, University College, Cardiff, has prepared students for the Preliminary Scientific examination of the University of London, and for the corresponding examinations of other licensing bodies. In 1893, Chairs of Anatomy and Physiology and a Lectureship in Materia Medica and Pharmacy were established, making it possible to spend three out of the five years of the medical curriculum at this school.

Arrangements have been made with the managing committee of the Cardiff Infirmary to give students of the college the privilege of attending this hospital, which contains 180 beds.

The composition fee for the three years' course of study for the Preliminary Scientific and Intermediate Examination for the London M.B. is £57 10s.

### University of Durham (College of Medicine, Newcastle-upon-Tyne).

For students who intend to graduate at the Durham University it is necessary that one of the five years of professional education should be spent in attendance at the College of Medicine, Newcastle-upon-Tyne. During the year so spent the student must attend the medical and surgical practice and clinical lectures at the Infirmary, which contains 280 beds. No residence at Durham is necessary. The remaining four years of the curriculum may be spent at Newcastle-upon-Tyne or at one or more of the recognised medical schools.

A new wing has been added to the College of Medicine to accommodate the departments of physiology and bacteriology. It also contains a students' gymnasium and a set of students' union rooms.

The New Royal Victoria Infirmary, containing 450 beds was recently opened by H.M. the King. In the new infirmary adequate accommodation will be provided for the study of the various special subjects in addition to the ordinary clinical work.

The composition fee for the complete course of lectures is 72 guineas, and for the medical and surgical practice of the hospital 25 guineas.

### The University of Leeds (School of Medicine).

Clinical instruction is given in the general infirmary, which contains 480 beds, and is amply provided with special departments.

Students of the University also have access for instruction to the Leeds Public Dispensary, the City Fever Hospitals, the Hospital for Women and Children, the Leeds Maternity Home, and the West Riding Asylum at Wakefield.

The School of Medicine, which is particularly well equipped in every way, is separated from the infirmary by the width of a street only, an arrangement which facilitates attendance at both institutions.

### The University of Liverpool (Faculty of Medicine).

Students are prepared for the degrees of the University of Liverpool, or may study for the degrees and qualifications of various other licensing bodies.

The laboratories are modern and very well equipped, and the facilities for instruction are most complete.

Courses of instruction in tropical diseases can be obtained at the Liverpool School of Tropical Medicine. Fellowships, scholarships, and prizes of over £800 are awarded annually.

The Clinical School of the University now consists of four general hospitals—the Royal Infirmary, the David Lewis Northern Hospital, the Royal Southern Hospital, and the Stanley Hospital; and of five special hospitals—the Eye and Ear Infirmary, the Hospital for Women, the Infirmary for Children, St. Paul's Eye and Ear Hospital, and St. George's Hospital for Skin Diseases. These hospitals contain in all a total of 1,127 beds. The organisation of these hospitals to form one teaching institution provides the medical student and the medical practitioner with a field for clinical education and study which is unrivalled in extent in the United Kingdom.

### The Victoria University of Manchester (Faculty of Medicine).

The medical department of the University is particularly well provided with facilities for teaching the preliminary subjects—namely, anatomy, physiology, pathology, and materia medica.

Clinical instruction is provided at the Manchester Royal Infirmary, which contains 290 beds, and also has large out-patient and casualty departments. Associated with the Infirmary is a Convalescent Hospital at Cheadle, containing 136 beds. Women students are admitted to the practice of the Infirmary on the same terms as men.

### Oxford University.

One of the chief events in the recent history of Oxford University has been the revival of the Medical School. Admirable arrangements now exist for instruction in the preliminary medical sciences; while a certain proportion of the clinical work can be done, if desired, in the wards of the Radcliffe Infirmary, previous to taking out the full course in the metropolis. Clinical lectures are given in medicine and surgery, and demonstrations in pathology, surgical anatomy, and medical and surgical diagnosis are given also. In order to obtain the Oxford medical degree it is necessary to be a graduate in Arts of the University.

### University of Sheffield (Faculty of Medicine).

The University is within easy distance of the Royal Infirmary (255 beds), the Royal Hospital (165 beds), the Jessop Hospital for Women, and the City Fever Hospital. It possesses numerous well-equipped laboratories and other necessaries for study and research. There is a large field for clinical work in the above-named hospitals.

The composition fee is £80 payable in three instalments, namely, £24 at commencement of first year, £28 at commencement of second year, £28 at commencement of third year, which admits to classes and courses of instruction required for the M.B., Ch.B. degrees of the University. This fee does not cover medical and surgical hospital practice and clinical work. Composition fee for medical and surgical hospital practice £36 15s. Temporary Hospital for Diseases of Women (80 beds) £3 3s., City Fever Hospitals (547 beds) £2 2s., South Yorkshire Asylum (1,610 beds) £2 2s.

---

### SCOTLAND.

### The Carnegie Trust and the Payment of Class Fees.

This is a fund to provide under certain regulations necessitous but deserving medical students with all or a portion of the class fees of the curriculum.

### University of Edinburgh.

To obtain the Edinburgh medical degree it is necessary to pass the Preliminary Examination or its recognised equivalent, and to pass four professional examinations. The first is in botany, zoology, physics, and chemistry; the second in anatomy, physiology, materia medica, and therapeutics; the third in pathology; and the final in surgery, clinical surgery, medicine, clinical medicine, midwifery, forensic medicine, and public health.

The degree of M.B. is not conferred separately from the Ch.B. In order to obtain the degree it is necessary to spend at least two of the five years of medical study in the University of Edinburgh.

Clinical instruction is given in the Royal Infirmary, which contains nearly 900 beds; Royal Hospital for Sick Children, with 120 beds; Maternity Hospital; City Fever Hospital; and the Royal Morningside Asylum.

A Diploma in Tropical Medicine and Hygiene has recently been instituted. It is conferred on graduates of the University after an approved course of study and examinations in Tropical diseases, bacteriology, medical entomology and protozoology, and clinical Tropical medicine.

### School of Medicine of the Royal Colleges, Edinburgh.

This school of medicine is composed of lecturers who lecture in several buildings near the Royal Infirmary. They are recognised or licensed by the Royal Colleges of Surgeons and Physicians, and also recognised by the University.

The teaching is similar to that of the Scottish Universities, and the students receive similar certificates at the close of each session. The lectures qualify for the University of Edinburgh and other Universities; the Royal Colleges of Physicians and Surgeons of Edinburgh, London, and Dublin; the Faculty of Physicians and Surgeons of Glasgow, and the other Medical Boards. The whole education required for graduation at the University of London may be taken in this school.

The facilities for clinical instruction are similar to those provided for the University. Full particulars and copy of the calendar of the school may be had gratis from the Secretary, R. N. Ramsay, solicitor, 27 Forrest Road, Edinburgh.

### Medical College for Women, Edinburgh.

Precisely the same facilities for medical study are given as to male students in the School of Medicine, Edinburgh. Regulations, fees, and arrangements are the same. The lecturers are recognised by the University of Edinburgh as giving courses admitting to the degrees of M.B., Ch.B. Clinical instruction is given in the Royal Infirmary and other hospitals mentioned above.

The benefit of the Carnegie Trust is open to students of the college.

### University of Glasgow.

The whole course of study for the M.B., Ch.B., can be passed in the Medical School of the University.

The regulations are similar to those given above for Edinburgh. Clinical instruction is given at the Western Infirmary, which contains 420 beds, and also at the Lunatic Asylum, the Eye Infirmary, the Maternity Hospital, the City Fever Hospitals, and various other hospitals.

The cost of the course, including matriculation, fees for classes, for hospital attendance, and for professional examinations, amounts to about £150.

### Queen Margaret College, Glasgow.
### (The Women's Department of the University).

This is an integral part of the University of Glasgow. The courses, regulations, and fees are the same as for men. The instruction is given by University Professors and Lecturers appointed by the University Court. The College provides a separate building, with class-rooms, laboratories, library, and other teaching appliances. The women have all the rights and privileges of University students, and do their clinical work in the Royal Infirmary, where special wards are reserved for their use, and in other hospitals.

### Anderson's College Medical School, Glasgow.

The college, at which a full medical curriculum can be obtained, is situated in Dumbarton Road, close to the Western Infirmary and within four minutes' walk of the University.

Degrees and diplomas: Certificates of attendance on the lectures are accepted by the Universities of London and Durham, by the Royal University of Ireland, and by all the Royal Colleges and licensing boards in the United Kingdom.

The Carnegie Trust extends its benefactions to students of Anderson's College. Particulars may be obtained from W. S. McCormick, Esq., LL.D., The Carnegie Trust Offices, Edinburgh.

### St. Mungo's College, Glasgow.

The college buildings, which are provided with class-room and laboratory accommodation for three or four hundred students, are situated within the grounds of the Royal Infirmary, the wards of which are available for clinical instruction, as also are those of the Western Infirmary. In connection with the former there are pathological and bacteriological laboratories, and a large pathological museum. All the classes in this college qualify for the diplomas of the English, Scottish, and Irish Conjoint Boards, and under certain conditions for the various universities.

### University of Aberdeen.

The regulations are practically the same as those given for Edinburgh. Clinical instruction is obtained in the Royal Infirmary, which contains 250 beds, in the Sick Children's Hospital, and several other institutions, including the Royal Lunatic Asylum and the City Fever Hospital.

---

### IRELAND

### Royal College of Surgeons in Ireland (The Schools of Surgery).

These schools are attached by charter to the Royal College of Surgeons in Ireland. They are carried on within the college buildings, and are specially subject to the supervision and control of the Council, who are empowered to appoint and remove the professors, and to regulate the methods of teaching pursued. The buildings have been reconstructed, the capacity of the dissecting-room nearly trebled, and special pathological, bacteriological, public health, chemical, and pharmaceutical laboratories fitted with the most approved appliances, in order that students may have the advantage of the most modern methods of instruction. There are special rooms set apart for lady students.

Summer session: Lectures and practical courses commence April 1; terminate June 30.

Winter session: Lectures and practical courses commence October 15; terminate March 31.

The medical students' guide will be forwarded post free on application to the Registrar, Royal College of Surgeons, Dublin.

### Queen's College, Belfast.

The courses of study meet the requirements of the Royal University of Ireland and other examining bodies.

Clinical instruction is given at the Royal Victoria Hospital and other hospitals.

Full information can be had on application to the Registrar, Queen's College, Belfast.

### Queen's College, Cork.

Students can attend courses which meet the requirements of the Royal University of Ireland, the Conjoint Boards of London, Edinburgh, or Dublin, and other examining bodies.

Clinical instruction is given at the North and South Infirmaries, each of which contains 100 beds, and at other hospitals.

Further information can be had on application to the Registrar, Queen's College, Cork.

The Medical School of the Catholic University, Ireland, Dublin.

Founded in 1855, it is now the largest medical school in Ireland. It prepares students specially for the examinations of the Royal University and the Conjoint Colleges of Ireland and Edinburgh, but its lectures and practical courses are recognised by all the licensing bodies in Great Britain and Ireland. In addition to the ordinary medical examinations it prepares students for the D.P.H. and for the various higher University examinations in pathology, physiology, chemistry, etc. Six exhibitions and numerous gold and silver medals are offered annually for competition. The school opens for dissections on October 1; the winter lectures begin on November 2. The summer session commences on April 1.

